# The fiery landscape of depression: A review of the inflammatory hypothesis

**DOI:** 10.12669/pjms.293.3357

**Published:** 2013

**Authors:** Ali Madeeh Hashmi, Muhammad Awais Aftab, Nauman Mazhar, Muhammad Umair, Zeeshan Butt

**Affiliations:** 1 Ali Madeeh Hashmi, MD, Foreign Professor III (HEC), Psychiatry,; 2 Muhammad Awais Aftab, MBBS, Graduate,; 3 Nauman Mazhar MBBS, MD, Assistant Professor, Psychiatry,; 4 Muhammad Umair, 4th Year MBBS,; 5 Zeeshan Butt, MBBS, Resident in Internal Medicine,

**Keywords:** Anti-depressants, Cytokines, Depression, Hypothalamic-pituitary-adrenal axis, Inflammation

## SUMMARY

The purpose of this article is to review the evidence linking depression with inflammation, to examine the bi-directional relationship between the neuro-humeral circuitry of depression and the inflammatory response, and point out new treatment implications of these ideas. The evidence available is in areas of genetic links, association of depression with raised inflammatory markers such as Tumour Necrosis Factor (TNF)-alpha, Interleukin (IL)-1, IL-6, co-morbidity of depression with inflammatory medical illnesses, administration of cytokines leading to depression, and the recognition that anti-depressants have anti-inflammatory and neuro-protective properties. Inflammatory response and mood regulation constitute a system of bi-directional communication such that inflammatory cytokines can penetrate the CNS and influence behavior. Activation of the CNS cytokine network leads to a cascade of effects such as disturbed metabolism of amino acids, neurotoxicity, diminished neurotrophic support, decreased neurogenesis, impaired negative feedback regulation of HPA axis function and glucocorticoid resistance. Treatment implications include strategies to screen for patients with increased inflammatory activity, possible treatment with anti-inflammatory agents, and the recognition of new target areas for antidepressant medications.

***Methodology: ***A literature search for articles published during the last ten years was conducted using various combinations of key words (‘depression’, ‘inflammation’, ‘cytokines’, ‘immune system’, ‘interleukins’) utilizing the databases Google Scholar and PubMed. An outline of the most relevant aspects of the role of inflammatory processes in depression was created. Major papers, including other review articles, were identified in accordance with our outline. These articles were subsequently hand searched and reviewed individually for further references of significance.

***Conclusion: ***The inflammatory response leading to formation of inflammatory cytokines plays a significant role in the pathophysiology of depression, and this has important implications with regards to new and personalized treatment of depression.

## INTRODUCTION

“O melancholy!

Whoever yet could sound thy bottom?”

**William Shakespeare**,* Cymbeline (1611)*

Depression is a condition that is probably as old as mankind and its underlying mechanism remains a subject of great dispute. At the start of the 20^th^ century there were two opposing tendencies within the psychiatric community: Emil Kraepelin viewed depression as a disease within the medical model and Sigmund Freud thought of it as a manifestation of psychodynamic processes.^1^ In the 1950s and 60s with the development of anti-depressant medications, the monoamine hypothesis and serotonin hypothesis came to dominate psychiatric research.^2^^,^^3^ While being simple and elegant, the monoamine hypothesis ran into several difficulties, ranging from lack of universal response to therapeutic latency period. Experimental attempts to confirm the monoamine hypothesis faced difficult methodological problems, and produced no consistent results.^4^^,^^5^

Amidst this background, attention has slowly shifted towards inflammatory (cytokine) hypothesis of depression in the last two decades. This hypothesis was first proposed by Smith in 1991 in the form of the ‘macrophage theory of depression’.^6^

To diagnose a case of Major Depressive Disorder (MDD), five of the following DSM-IV symptoms are required to be present for a minimum period of 2-weeks: (i) depressed mood; (ii) loss of interest or pleasure; (iii) significant weight or appetite alteration; (iv) insomnia or hypersomnia; (v) psychomotor agitation or retardation; (vi) fatigue and loss of energy; (vii) feelings of worthlessness or guilt; (viii) diminished ability to think or concentrate or indecisiveness; and (ix) suicidal ideation.^7^

## METHODOLOGY

For the purpose of this review, a literature search for articles published during the last ten years was conducted using various combinations of key words (‘depression’, ‘inflammation’, ‘cytokines’, ‘immune system’, ‘interleukins’) utilizing the databases Google Scholar and PubMed. An outline of the most relevant aspects of the role of inflammatory processes in depression was created. Major papers, including other review articles, were identified in accordance with our outline. These articles were subsequently hand searched and reviewed individually for further references of significance.

***Sickness Behavior and Evolutionary Background:***

The body has a very organized strategy to combat infection, and this is manifested not just in fever and other physiological changes, but also in behavioral and affective alterations. The result is so-called sickness behavior, an adaptive set of behaviors induced by infection and inflammation consisting of lethargy, depression, loss of appetite, sleepiness and reduction in grooming.^8^ The purpose of this is to conserve the energy resources of the sick individual and help fight back infection. Pro-inflammatory cytokines, produced by the activated innate immune system in response to specific Pathogen-Associated Molecular Patterns (PAMP), trigger sickness behavior. These cytokines include Interleukin (IL) 1, IL-6 and tumor necrosis factor α (TNF-α).^8^ It has long been recognized that there are significant overlaps between sickness behavior and depression, such as anhedonia, decreased appetite, disturbed sleep, decreased activity and social withdrawal.^9^ It may well be the case that depression is an evolutionary psychological byproduct of early mechanisms that promoted diversion of energy sources towards fighting the infection.^10^ The dysregulation of glucocorticoid responses that has been observed in depression may have been of further advantage in this regard.^11^ Before the modern human practices of hygiene and later development of antimicrobial agents, genes promoting sickness behavior would have been crucial in providing a survival advantage that would come at the cost of the risk of depression. It may be hypothesized that depressive mood disorder is an off-shoot of the genetic inflammatory machinery, which was otherwise of huge benefit in the pre-antibiotic era.^12^^,^^13^

## A REVIEW OF THE EVIDENCE

***1) Genetic Links between Inflammation and Depression:***

There have been several discoveries of associations between genes related to inflammation and depression. Jun TY et al reported that patients of Major Depressive Disorder had an increased frequency of the TNF2 (A) allele, suggesting that tumour necrosis factor-alpha gene polymorphism may play some role in the susceptibility for depression.^14^ In another study by Wong ML et al, single nucleotide polymorphisms (SNPs) in two genes critical for T-cell function were found to be associated with predisposition for MDD. These two genes were responsible for antigen processing and differentiation. Furthermore, a significant combined allele dose-effect was revealed such that the likelihood of MDD increased with the number of alleles.^15^ Revealing further genetic linkages, in a study by Yu YW et al it was observed that all patients of MDD who were homozygous for the -511T allele of the IL-1beta gene had less severity of depressive symptoms and more favorable Fluoxetine response compared to -511C carriers.^16^

***2) Association of Depression with raised inflammatory markers:***

The discovery that depression is associated with raised inflammatory markers was an early finding. A meta-analysis by Dowlati Y et al reported significantly higher concentrations of the proinflammatory cytokines TNF-α and IL-6 in patients of depression compared with control subjects. While individual studies have reported variable results both in favor and against, the meta-analysis strengthened the case in favor of depression’s association with inflammatory response system.^17^ This is supported by other studies such as a meta-analytic review reporting increased circulating IL-6 levels in depression^18^, and the WFSBP Task Force on Biological Markers study revealing a robust association with increase of soluble interleukin-2 receptor and interleukin-6 in serum, and impaired suppression of the dexamethasone suppression test.^19^

***3) Association of Co-morbid Depression with Inflammatory Medical Illnesses:***

As the role of inflammatory processes in the etiology of diseases such as diabetes^20^, cardiovascular events^21^ and cancer^22^ was being recognized, the high co-morbid rates of depression in these disease provided an indirect evidence for the inflammatory hypothesis of depression.^23^ We have reports of high co-morbid depression in inflammatory diseases such as Rheumatoid Arthritis^24^, Fibromyalgia^25^, Inflammatory Bowel Disease^26^ and Coronary artery disease.^27^ Depression co-morbidity has also been reported in neurodegenerative disorders^28^ such as Parkinson's and Alzheimer's diseases in which recent studies have also elaborated significant neuro-inflammation.^29^^-^^31^

***4) Administration of Cytokines leads to Depression:***

In many experimental studies carried out on animals, administration of cytokines has been demonstrated to produce depressive behavior in non-human primates such as monkeys.^32^ Administration of TNF-α in mice has been shown to induce depressive behavior, which is attenuated by administration of anti-depressants.^33^ In such studies, the relationship between the inflammatory marker and the response in the form of depressive behavior were followed up after the administration of the inflammatory mediators. In non-human primates, administration of a plethora of inflammatory cytokines was possible in different experimental settings. Such experiments cannot be conducted on human beings due to obvious ethical reasons; however, the clinical follow ups of hepatitis C patients undergoing treatment with interferon have provided significant data for humans. One study of HCV patients undergoing treatment reported a negative correlation of log-transformed CSF concentrations of IL-6 with log-transformed CSF 5-HIAA, a serotonin metabolite, which was found to be the strongest predictor of depressive symptoms in the study.^34^Another study investigating interferon (IFN) therapy in patients with hepatitis C (HCV) revealed that 33% of patients developed IFN-induced Major Depressive Disorder, 85% of which were responsive to antidepressant treatment.^35^Another study found significantly increased scores for depression (p <.001) and anger/hostility (p <.001) during IFN alpha therapy in the treatment group compared with the untreated reference group.^36^ Furthermore, in patients with malignant melanoma, pretreatment with Paroxetine appears to be an effective strategy for minimizing depression induced by IFN alpha.^37^

***5) Anti-Depressants have anti-inflammatory and neuro-protective properties:***

The anti-inflammatory and neuro-protective role of anti-depressants is increasingly being recognized. Hwang J et al investigated the effects of tricyclic antidepressants using cultured brain cells as models. Their results showed that Clomipramine and Imipramine significantly decreased the production of nitric oxide and tumor necrosis factor-alpha (TNF-a) in microglia and astrocyte cultures. Furthermore, the expression of pro-inflammatory cytokines was attenuated at mRNA levels. In the same study Clomipramine and Imipramine were shown to be neuroprotective as the drugs reduced microglia-mediated neuroblastoma cell death in the microglia/ neuron co-culture.^38^

Similar results have also been demonstrated with studies involved Fluoxetine^39^ as well as Paroxetine and Sertraline.^40^ In a meta-analysis of human studies where cytokine levels were measured in patients of major depression before and after treatment with anti-depressants, it was demonstrated that IL-1β and possibly IL-6 levels were reduced, adding to the evidence regarding anti-inflammatory effects of anti-depressants.^41^

## A REVIEW OF PATHOPHYSIOLOGIC MECHANISMS

The brain was previously considered to be an ‘immune-privileged’ organ but recent work has shown this conception to be mistaken.^42^^,^^43^ The brain not only has inflammatory cells of its own (macrophages, microglia and dendritic cells) and possesses receptors for inflammatory mediators; peripheral inflammatory factors can also influence brain’s functioning.

***Cytokines and Blood-Brain Barrier:***

There are a number of mechanisms by which peripheral cytokines can gain access to and/or influence central neural activity, as elaborated by Raison CL et al.^13^ One pathway is via macrophage-like cells in the circumventricular organs and the choroid plexus (lying outside the blood-brain barrier), which detect and respond to circulating pathogen-associated molecular patterns by producing pro-inflammatory cytokines. These cytokines then cross the blood-brain barrier by volume diffusion.^44^^,^^45^ A second mechanism of cytokine entry into brain is via cytokine transporters at the blood–brain barrier^46^ Perivascular macrophages and endothelial cells of brain venules have been shown to possess IL-1 receptors which respond by producing local prostaglandin E2, constituting a third pathway^47^^,^^48^and lastly, activation of vagal afferent fibres has also been shown to communicate cytokine signals to various brain nuclei.^49^

***Central Nervous System (CNS) Cytokine Network:***

CNS has a network of immune cells (microglia) which produce cytokines, have cytokine receptors, amplify cytokine signals and influence neurotransmitter metabolism in brain areas, including those concerned with emotions and reward.^10^^,^^13^ Moreover, these inflammatory cytokines have significant stimulatory effects on brain CRH production and HPA axis hormones.^50^^-^^52^

***Neurotransmitter Metabolism and Neurotoxicity:***

Central to the cytokine effects on amino acid metabolism is the enzyme, Indoleamine 2,3Dioxygenase (IDO). Cytokines lead to activation of IDO through multiple signaling pathways, including Nuclear Factor Kappa-light-chain-enhancer of activated B cells(NF-κB),Mitogen-Activated Protein Kinase (MAPK) and Signal Transducer and Activator of Transcription 5 (STAT5).^53^ IDO breaks down Tryptophan, the precursor of Serotonin, into Kynurenine (KYN), resulting in a reduction in the level of Serotonin.^54^ Reduction of Serotonin is associated with depression, as has long been known, but depression resulting from IDO activation and KYN production has additional Serotonin-independent effects as well. For instance, depressive behavior in mice has been demonstrated with administering KYN alone.^55^

KYN is further converted into Quinolinic acid (QUIN) in microglia.^54^ QUIN promotes glutamate release through activation of N-methyl-D-aspartate (NMDA) receptors as well as producing oxidative stress, the combination of which leads to neurotoxicity.^56^

***Diminished Neurotrophic Support and Decreased Neurogenesis:***

The CNS Cytokine network has also been shown to exert effects on neurotrophic support and neurogenesis, shedding new light on the pathophysiology of depression. Prominently, inflammatory cytokines hinder neuronal synaptic plasticity, decrease the levels of neurotrophic factors especially Brain Derived Neurotrophic Factor (BDNF), and reduce neurogenesis in the hippocampus.^57^^-^^59^ Decreased levels of BDNF have also been reported to be a robust biological marker of major depression.^19^ In support of this, Serotonin Specific Reuptake Inhibitors (SSRIs-the most commonly used antidepressants) have been shown to increase neurogenesis in the hippocampus^60^ and potentiate the effects of BDNF.^61^ In this context of decreased neurotrophic support, the neurotoxic effects of glutamate, as were noted previously, are further enhanced.

***Hypothalamic-Pituitary-Adrenal (HPA) Axis and Glucocorticoid Resistance:***

Cytokines have been shown to increase the levels of corticotropin-releasing hormone (CRH), adrenocorticotropic hormone (ACTH), and cortisol. These hormones have also been reported in studies to be elevated in patients of depression.^62^^,^^63^

The pathway for this appears to be impaired negative feedback regulation of HPA axis function and glucocorticoid resistance (determined by the dexamethasone suppression test). This is also, in part, related to reduced glucocorticoid receptor (GR) expression.^63^^,^^64^ Cytokine signaling molecules such as NF-κB, MAPK, and signal transducer and activator of transcription 5 (STAT5) have been demonstrated to inhibit GR.^65^

As would be expected, antidepressants have been shown to restore the negative feedback control of HPA axis and increase GR expression.^58^

***Psychological Stress Activates Pro-Inflammatory Cytokines:***

In cases of depression which occur in patients of various medical diseases, the source of inflammation is clear enough. One wonders what triggers the inflammation in patients of depression who are otherwise medically healthy. Recent work in this regard reveals that psychological stress can activate pro-inflammatory cytokines (such as IL-1 and TNF-α) and decrease anti-inflammatory cytokines (such as IL-10). This has been demonstrated in animals as well as humans, with both acute and chronic stress.^66^^-^^70^ In addition, psychosocial stress can also activate NF-κB.^71^Psychosocial stress induced activation of NF-κB may also possibly occur via stimulation of sympathetic nervous system.^72^

**Fig.1:**
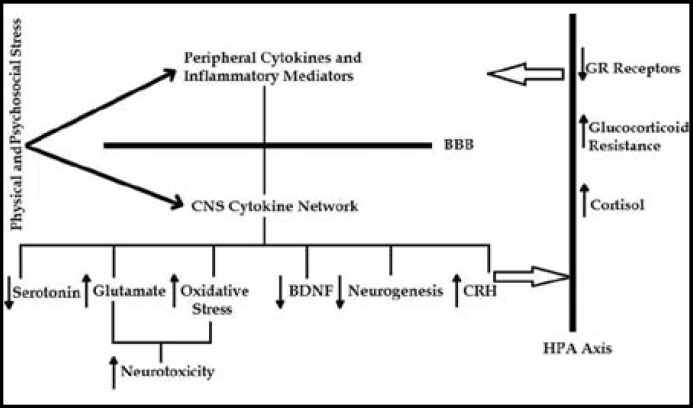
Bi-directional relationship between neuro-humeral circuitry of Depression and Inflammatory response

## TREATMENT IMPLICATIONS

***Screening Strategies:***

The use of inflammatory biomarkers can potentially be used as a strategy to screen for patients with increased inflammatory activity who are less likely to respond to conventional anti-depressant therapy. Such patients may be more likely to benefit from inflammation targeted therapies.^13^^,^^73^

***Possible Treatment with Anti-inflammatory agents:***

Various studies have reported increased efficacy of treatment with the addition of anti-inflammatory agents to anti-depressants. Addition of Celecoxib to Sertraline^74^,Fluoxetine^75^ and Reboxetine^76^ is of benefit with greater reduction of inflammatory markers and severity of depressive symptoms than either of the anti-depressant alone. Addition of Acetylsalicylic acid to Fluoxetine has also been shown to increase remission rates.^77^

Usage of antagonists of TNF-α (Etanercept, Infliximab) without anti-depressants in the context of patients suffering from autoimmune disorders has also been shown to decrease the severity of depression.^78^^,^^79^

However, at the moment, no clinical recommendations can be made with regards to the use of anti-inflammatory agents in the treatment of depression, considering that current anti-inflammatory drugs often have serious side-effects of their own. Celecoxib, for instance, is associated with adverse and potentially fatal cardiovascular thrombotic events.^80^ Further research and development of possibly new anti-inflammatory agents specific to depression is required before any suggestions can be made regarding their clinical use.

***New Target Areas for Medications:***

The mechanism of how inflammation leads to depression provides many new target areas for researchers to explore in developing new medications. These target areas include cytokines and their signaling pathways (including NF-κB), CNS immune cells, such as microglia, NMDA Glutamate antagonists and neurotrophic factors.

***Special Considerations for Pakistan:***

Pakistan, like many ‘third world’ countries, suffers from high rates of chronic inflammatory illness. Two common ones include Tuberculosis^81^ and Hepatitis C^82^. Given the high prevalence of these illnesses in Pakistan and the fact that both these illnesses would be expected to cause serious derangement of the immunomodulatory response, the review and recommendations above have a special significance for Pakistan. A number of studies from Pakistan have reported the association of depression with inflammatory medical conditions, including Hepatitis^83^, Tuberculosis^84^, Psoriasis Vulgaris^85^, Rheumatologic disorders^86^, Coronary artery disease^87^, Chronic pediatric illnesses^88^ and Dengue Fever.^89^ Keeping in mind the evidence that has so far accumulated regarding the link between the inflammatory immune response and depression, increased vigilance and regular screening is required for early detection and treatment of depression in patients with chronic inflammatory illness (such as the above). Untreated depression in these patients can lead to non-compliance, partial or non-response to treatment and increased morbidity and mortality.

## CONCLUSION

An impressive body of evidence has accumulated over the last two decades linking depression with inflammation, leading to the development of an inflammatory (cytokine) hypothesis of depression. Inflammatory response and mood regulation constitute a system of bi-directional communication such that inflammatory response leading to the formation of inflammatory cytokines can penetrate the CNS and influence behavior in the form of depression and sickness behavior, and mood regulatory system can potentially modify the immune system via the hypothalamus pituitary adrenal axis. Treatment implications include strategies to screen for patients with increased inflammatory activity who are less likely to respond to conventional anti-depressant therapy, possible treatment with anti-inflammatory agents, and the recognition of new target areas for researchers in developing new antidepressant medications.

## Author’s contribution

Ali Madeeh Hashmi was involved in conception of the study, literature review, manuscript writing and final approval. Muhammad Awais Aftab contributed to outline of the study, literature search and review, drafting the article and final revision. Nauman Mazhar contributed significantly to the revisions and rewriting and also provided suggestions regarding additional references for the rewrites and revisions. Substantial contributions to literature search and early drafting were provided by Muhammad Umair and Zeeshan Butt.

## References

[1] Brigitta B (2002). Pathophysiology of depression and mechanisms of treatment. Dialogues Clin Neurosci.

[2] Schildkraut JJ (1965). The catecholamine hypothesis of affective disorders: A review of supporting evidence. J Neuropsychiatry Clin Neurosci.

[3] Coppen A (1967). The biochemistry of affective disorders. Br J Psychiatry.

[4] Roggenbach J, Muller-Oerlinghausen B, Franke L (2002). Suicidality, impulsivity, and aggression-Is there a link to 5HIAA concentration in the cerebrospinal fluid. Psychiatry Res.

[5] Heninger G, Delgado P, Charney D (1996). The revised monoamine theory of depression: A modulatory role for monoamines, based on new findings from monoamine depletion experiments in humans. Pharmacopsychiatry.

[6] Smith RS (1991). The macrophage theory of depression. Med Hypotheses.

[7] (1994). Diagnostic and Statistical Manual of Mental Disorders IV.

[8] Dantzer R (2006). Cytokine, sickness behavior, and depression. Neurol Clin.

[9] Dantzer R (2004). Cytokine-induced sickness behaviour: a neuroimmune response to activation of innate immunity. Eur J Pharmacol.

[10] Dunn AJ (1999). Effects of cytokines on cerebral neurotransmission Comparison with the effects of stress. AdvExp Med Biol.

[11] Avitsur R (2001). Social stress induces glucocorticoid resistance in subordinate animals. Horm Behav.

[12] Maier SF, Watkins LR (1998). Cytokines for psychologists: implications of bidirectional immune-to-brain communication for understanding behavior, mood, and cognition. Psychol Rev.

[13] Raison CL, Capuron L, Miller AH (2006). Cytokines sing the blues: inflammation and the pathogenesis of depression. Trends Immunol.

[14] Jun TY (2003). Possible association between -G308A tumour necrosis factor-alpha gene polymorphism and major depressive disorder in the Korean population. Psychiatr Genet.

[15] Wong ML, Dong C, Maestre-Mesa J, Licinio J (2008). Polymorphisms in inflammation-related genes are associated with susceptibility to major depression and antidepressant response. Mol Psychiatry.

[16] Yu YW (2003). Association study of the interleukin-1 beta (C-511T) genetic polymorphism with major depressive disorder, associated symptomatology, and antidepressant response. Neuropsychopharmacology.

[17] Dowlati Y, Herrmann N, Swardfager W, Liu H, Sham L, Reim EK (2010). A meta-analysis of cytokines in major depression. Biol Psychiatry.

[18] Zorilla EP, Luborsky L, McKay JR, Roesnthal R, Houldin A, Tax A (2001). The relationship of depression and stressors to immunological assays: A meta-analytic review. Brain Behav Immun.

[19] Mossner R, Mikova O, Koutsilieri E, Saoud M, Ehlis AC, Muller N (2007). Consensus paper of the WFSBP Task Force on Biological Markers: Biological markers in depression. World J Biol Psychiatry.

[20] Wellen KE, Hotamisligil GS (2005). Inflammation, stress, and diabetes. J Clin Invest.

[21] Willerson JT, Ridker PM (2004). Inflammation as a cardiovascular risk factor. Circulation.

[22] Li Q (2005). Inflammation-associated cancer: NF-kappaB is the lynchpin. Trends Immunol.

[23] Evans DL (2005). Mood disorders in the medically ill: scientific review and recommendations. Biol Psychiatry.

[24] Bruce TO (2008). Comorbid depression in rheumatoid arthritis: Pathophysiology and clinical implications. Current Psychiatry Reports.

[25] Thieme K, Turk DC, Flor H (2004). Comorbid Depression and Anxiety in Fibromyalgia Syndrome: Relationship to Somatic and Psychosocial Variables. Psychosomatic Medicine.

[26] Graff LA, Walker JR, Bernstein CN (2009). Depression and anxiety in inflammatory bowel disease: A review of comorbidity and management. Inflamm Bowel Dis.

[27] Frasure-Smith N, Lesperance F (2006). Depression and coronary artery disease. Herz.

[28] Anisman H, Merali Z, Hayley S (2008). Neurotransmitter, peptide and cytokine processes in relation to depressive disorder: Comorbidity between depression and neurodegenerative disorders. Progress in Neurobiology.

[29] Wheeler RD, Owens T (2005). The changing face of cytokines in the brain: perspectives from EAE. Curr Pharm Des.

[30] Czlonkowska A, Kurkowska-Jastrzebska I, Czlonkowski A, Peter D, Stefano GB (2002). Immune processes in the pathogenesis of Parkinson's disease - a potential role for microglia and nitric oxide. Med Sci Monit.

[31] Cotter RL, Burke WJ, Thomas VS, Potter JF, Zheng J, Gendelman HE (1999). Insights into the neurodegenerative process of Alzheimer's disease: a role for mononuclear phagocyte-associated inflammation and neurotoxicity. J Leukoc Biol.

[32] Felger JC, Alagbe O, Hu F, Mook D, Freeman AA, Sanchez MM (2007). Effects of interferon-alpha on rhesus monkeys: a nonhuman primate model of cytokine-induced depression. Biol Psychiatry.

[33] Kaster MP, Gadotti VM, Calixto JB, Santos AR, Rodrigues AL (2012). Depressive-like behavior induced by tumor necrosis factor-alpha in mice. Neuropharmacology.

[34] Raison CL, Borisov AS, Majer M, Drake DF, Pagnoni G, Woolwine BJ (2009). Activation of central nervous system inflammatory pathways by interferon-alpha: relationship to monoamines and depression. Biol Psychiatry.

[35] Hauser P, Khosla J, Aurora H, Laurin J, Kling MA, Hill J (2002). A prospective study of the incidence and open-label treatment of interferon-induced major depressive disorder in patients with hepatitis C. Mol Psychiatry.

[36] Kraus MR, Schafer A, Faller H, Csef H, Scheurlen M (2003). Psychiatric symptoms in patients with chronic hepatitis C receiving interferon alfa-2b therapy. J Clin Psychiatry.

[37] Musselman DL, Lawson DH, Gumnick JF, Manatunga AK, Penna S, Goodkin RS (2001). Paroxetine for the prevention of depression induced by high-dose interferon alfa. N Engl J Med.

[38] Hwang J, Zheng LT, Ock J, Lee MG, Kim SH, Lee HW (2008). Inhibition of glial inflammatory activation and neurotoxicity by tricyclic antidepressants. Neuropharmacology.

[39] Liu D, Wang Z, Liu S, Wang F, Zhao S, Hao A (2011). Anti-inflammatory effects of fluoxetine in lipopolysaccharide(LPS)-stimulated microglial cells. Neuropharmacology.

[40] Horikawa H, Kato TA, Mizoguchi Y, Monji A, Seki Y, Ohkuri T (2010). Inhibitory effects of SSRIs on IFN-gamma induced microglial activation through the regulation of intracellular calcium. Prog Neuropsychopharmacol Biol Psychiatry.

[41] Hannestad J, DellaGioia N, Bloch M (2011). The effect of antidepressant medication treatment on serum levels of inflammatory cytokines: a meta-analysis. Neuropsychopharmacology.

[42] Galea I, Bechmann I, Perry VH (2007). What is immune privilege (not)?. Trends Immunol.

[43] Dantzer R, O’Connor JC, Freund GG, Johnson RW, Kelley KW (2008). From inflammation to sickness and depression: when the immune system subjugates the brain. Nat Rev Neurosci.

[44] Quan N, Whiteside M, Herkenham M (1998). Time course and localization patterns of interleukin-1beta messenger RNA expression in brain and pituitary after peripheral administration of lipopolysaccharide. Neuroscience.

[45] Vitkovic L (2000). Cytokine signals propagate through the brain. Mol Psychiatry.

[46] Banks WA (2006). The blood-brain barrier in psychoneuroimmunology. Neurol Clin.

[47] Konsman JP, Vigues S, Mackerlova L, Bristow A, Blomqvist A (2004). Rat brain vascular distribution of interleukin-1 type-1 receptor immunoreactivity: relationship to patterns of inducible cyclooxygenase expression by peripheral inflammatory stimuli. J Comp Neurol.

[48] Schiltz JC, Sawchenko PE (2002). Distinct brain vascular cell types manifest inducible cyclooxygenase expression as a function of the strength and nature of immune insults. J Neurosci.

[49] Bluthe RM (1994). Lipopolysaccharide induces sickness behaviour in rats by a vagal mediated mechanism. C R Acad Sci III.

[50] Besedovsky HO, Del Rey A (1996). Immune-neuro-endocrine interactions: facts and hypotheses. Endocr Rev.

[51] Silverman MN (2005). Immune modulation of the hypothalamic-pituitary-adrenal (HPA) axis during viral infection. Viral Immunol.

[52] Capuron L, Miller AH (2004). Cytokines and psychopathology: lessons from interferon-alpha. Biol Psychiatry.

[53] Fujigaki H, Saito K, Fujigaki S, Takemura M, Sudo K, Ishiguro H (2006). The signal transducer and activator of transcription 1 alpha and interferon regulatory factor 1 are not essential for the induction of indoleamine 2,3-dioxygenase by lipopolysaccharide: Involvement of p38 mitogen-activated protein kinase and nuclear factor-kappaB pathways, and synergistic effect of several proinflammatory cytokines. J Biochem.

[54] Schwarcz R, Pellicciari R (2002). Manipulation of brain kynurenines: Glial targets, neuronal effects, and clinical opportunities. J Pharmacol Exp Ther.

[55] O'Connor JC, Lawson MA, Andre C, Moreau M, Lestage J, Castanon N (2009). Lipopolysaccharide-induced depressive-like behavior is mediated by indoleamine 2,3-dioxygenase activation in mice. Mol Psychiatry.

[56] McNally L, Bhagwagar Z, Hannestad J (2008). Inflammation, glutamate, and glia in depression: A literature review. CNS Spectr.

[57] Wu CW, Chen YC, Yu L, Chen HI, Jen CJ, Huang AM (2007). Treadmill exercise counteracts the suppressive effects of peripheral lipopolysaccharide on hippocampal neurogenesis and learning and memory. J Neurochem.

[58] Antonioli M, Rybka J, Carvalho LA (2012). Neuroimmune endocrine effects of antidepressants. Neuropsychiatr Dis Treat.

[59] Song JH, Marszalec W, Kai L, Yeh JZ, Narahashi T (2012). Antidepressants inhibit proton currents and tumor necrosis factor-alpha production in BV2 microglial cells. Brain Res.

[60] Wang Y, Cui XL, Liu YF, Gao F, Wei D, Li XW (2011). LPS inhibits the effects of fluoxetine on depression-like behavior and hippocampal neurogenesis in rats. Prog Neuro-Psychopharmacology Biol Psychiatry.

[61] Chiou SH, Chen SJ, Peng CH, Chang YL, Ku HH, Hsu WM (2006). Fluoxetine up-regulates expression of cellular FLICE-inhibitory protein and inhibits LPS-induced apoptosis in hippocampus-derived neural stem cell. Biochem Biophysical Res Communications.

[62] Besedovsky HO, del Rey A (1996). Immune-neuro-endocrine interactions: Facts and hypotheses. Endocr Rev.

[63] Pariante CM, Miller AH (2001). Glucocorticoid receptors in major depression: Relevance to pathophysiology and treatment. Biol Psychiatry.

[64] Pace TW, Miller AH (2009). Cytokines and glucocorticoid receptor signaling Relevance to major depression. Ann N Y Acad Sci.

[65] Pace TW, Hu F, Miller AH (2007). Cytokine-effects on glucocorticoid receptor function: Relevance to glucocorticoid resistance and the pathophysiology and treatment of major depression. Brain Behav Immun.

[66] Madrigal JL (2002). The increase in TNF-αlpha levels is implicated in NF-kappaB activation and inducible nitric oxide synthase expression in brain cortex after immobilization stress. Neuropsychopharmacology.

[67] O’Connor KA (2003). Peripheral and central proinflammatory cytokine response to a severe acute stressor. Brain Res.

[68] Goebel MU (2000). Interleukin-6 and tumor necrosis factor-alpha production after acute psychological stress, exercise, and infused isoproterenol: differential effects and pathways. Psychosom Med.

[69] Deinzer R (2004). Acute stress effects on local Il-1beta responses to pathogens in a human in vivo model. Brain Behav Immun.

[70] Maes M (1998). The effects of psychological stress on humans: increased production of pro-inflammatory cytokines and a Th1-like response in stress-induced anxiety. Cytokine.

[71] Bierhaus A (2003). A mechanism converting psychosocial stress into mononuclear cell activation. Proc. Natl. Acad. Sci. U. S. A.

[72] Bierhaus A, Wolf J, Andrassy M, Rohleder N, Humpert PM, Petrov D (2003). A mechanism converting psychosocial stress into mononuclear cell activation. Proc Natl Acad Sci USA.

[73] Miller AH, Maletic V, Raison CL (2009). Inflammation and its discontents: the role of cytokines in the pathophysiology of major depression. Biol Psychiatry.

[74] Abbasi SH, Hosseini F, Modabbernia A, Ashrafi M, Akhondzadeh S (2012). Effect of celecoxib add-on treatment on symptoms and serum IL-6 concentrations in patients with major depressive disorder: Randomized double-blind placebo-controlled study. J Affect Disord.

[75] Akhondzadeh S, Jafari S, Raisi F, Nasehi AA, Ghoreishi A, Salehi B (2009). Clinical trial of adjunctive celecoxib treatment in patients with major depression: a double blind and placebo controlled trial. Depression Anxiety.

[76] Muller N, Schwarz MJ, Dehning S, Douhe A, Cerovecki A, Goldstein-Muller B (2006). The cyclooxygenase-2 inhibitor celecoxib has therapeutic effects in major depression: Results of a double-blind, randomized, placebo controlled, add-on pilot study to reboxetine. Mol Psychiatry.

[77] Mendlewicz J, Kriwin P, Oswald P, Souery D, Alboni S, Brunello N (2006). Shortened onset of action of antidepressants in major depression using acetylsalicylic acid augmentation: A pilot open-label study. Int Clin Psychopharmacol.

[78] Lichtenstein GR (2002). Infliximab improves quality of life in patients with Crohn’s disease. Inflamm Bowel Dis.

[79] Mathias SD (2000). Health-related quality of life and functional status of patients with rheumatoid arthritis randomly assigned to receive etanercept or placebo. Clin Therap.

[80] Trelle S, Reichenbach S, Wandel S, Hildebrand P, Tschannen B, Villiger PM (2011). Cardiovascular safety of non-steroidal anti-inflammatory drugs: network meta-analysis. BMJ.

[81] Vermund SH, Altaf A, Samo RN, Khanani R, Baloch N, Qadeer E (2009). Tuberculosis in Pakistan: A decade of progress, a future of challenge. J Pak Med Assoc.

[82] Umar M, Bushra H, Ahmad M, Khurram M, Usman S, Arif M (2010). Hepatitis C in Pakistan: a review of available data. Hepat Mon.

[83] Majeed S, Memon A, Abdi MA (2009). Frequency of depression among Hepatitis C patients. Kust Med J.

[84] Aamir S (2010). Co-morbid anxiety and depression among pulmonary tuberculosis patients. J Coll Physicians Surg Pak.

[85] Nasreen S, Ahmed I, Effendi S (2008). Frequency and magnitude of anxiety and depression in patients with Psoriasis Vulgaris. J Coll Physicians Surg Pak Jul.

[86] Waheed A, Hameed K, Khan AM, Syed JA, Mirza AI (2006). The burden of anxiety and depression among patients with chronic rheumatologic disorders at a tertiary care hospital clinic in Karachi, Pakistan. J Pak Med Assoc.

[87] Bokhari SS, Samad AH, Hanif S, Hadique S, Cheema MQ, Fazal MAS (2002). Prevalence of depression in patients with coronary artery disease in a tertiary care hospital in Pakistan. J Pak Med Assoc.

[88] Taj R, Sikander KS, Khan AM (2002). Depression and Anxiety disorders in Children with Chronic Medical Problems and Healthy Children. J Coll Physicians Surg Pak.

[89] Hashmi AM, Butt Z, Idrees Z, Niazi M, Yousaf Z, Haider SF (2012). Anxiety and Depression Symptoms in Patients with Dengue Fever and Their Correlation with Symptom Severity. Int J Psychiatry Med.

